# A Case of Bilateral Hemorrhagic Pleural Effusion Due to Dual Antiplatelet Therapy in a Dialysis Patient

**DOI:** 10.7759/cureus.24450

**Published:** 2022-04-24

**Authors:** Tahmina Jahir, Sadaf Hossain, Tsering Dolkar, Meet J Patel, Ruby Risal, Ahmad Khan, Aneeta Kumari, Marie Schmidt, Danilo Enriquez, Harish Patel

**Affiliations:** 1 Pulmonary and Critical Care Medicine, One Brooklyn Health System-Interfaith Medical Center, New York, USA; 2 Internal Medicine, Jamaica Hospital Medical Center, New York, USA

**Keywords:** spontaneous hemothorax, hemothorax, fibrothorax, cardiac stent, dual anti-platelet therapy, dialysis, hemorrhagic pleural effusion

## Abstract

Etiologies of hemorrhagic pleural effusions (hemithoraces) are multifactorial. They can be traumatic, non-traumatic, or idiopathic in nature. In this report, we present a rare case of a 64-year-old male with end-stage renal disease (ESRD) on chronic hemodialysis and dual antiplatelet therapy (DAPT), due to a recent history of coronary arterial stent placement, who presented with progressive shortness of breath for one month. The CT of the chest revealed bilateral large pleural effusions (left > right) with a complete collapse of the left lung and partial collapse of the right lung. Ultrasound-guided left-sided thoracentesis revealed hemorrhagic pleural effusions. After the discontinuation of DAPT, drainage from the right-sided pleural effusion via a pigtail catheter showed continued drainage of pleural fluid without hemorrhage. The effusion on the left side was also noted to have resolved on the repeat chest X-ray. Prompt recognition of this rare cause of any hemorrhagic pleural effusion is essential for patients on dialysis to avoid complications. This report focuses on the possible etiology and potential complications of a hemorrhagic pleural effusion, followed by a brief discussion on the rare but significant association involving the incidence of a hemorrhagic pleural effusion in a dialysis patient receiving DAPT.

## Introduction

A pleural effusion is defined as the collection of fluid between the visceral and parietal pleura. The accumulation could occur due to disorders of the lungs, pleura, liver, kidneys, or heart, or it may be secondary to a systemic illness. Hence, it is important to identify the cause in order to guide the treatment. The etiology of a pleural effusion, however, remains unclear in nearly 20% of cases [1]. Hemothorax is defined as pleural fluid with a hematocrit of greater than or equal to 50% of the peripheral blood hematocrit. Pleural fluid with a red blood cell count of greater than 100,000 cells/µl is considered a hemorrhagic pleural effusion [2]. The etiology of hemorrhagic pleural effusion may be traumatic, iatrogenic, or non-traumatic; e.g., in end-stage renal disease (ESRD) patients, it may be caused by an underlying platelet dysfunction.

## Case presentation

**Figure 1:**
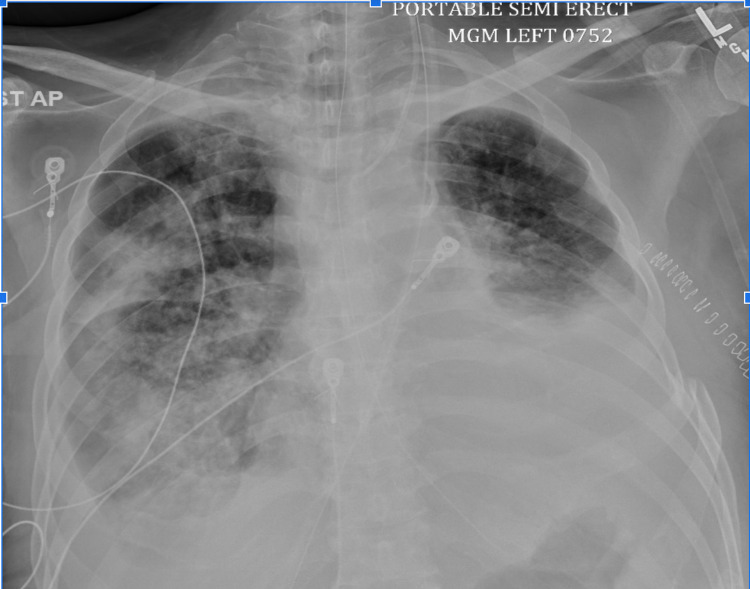
First chest X-ray on admission

**Figure 2:**
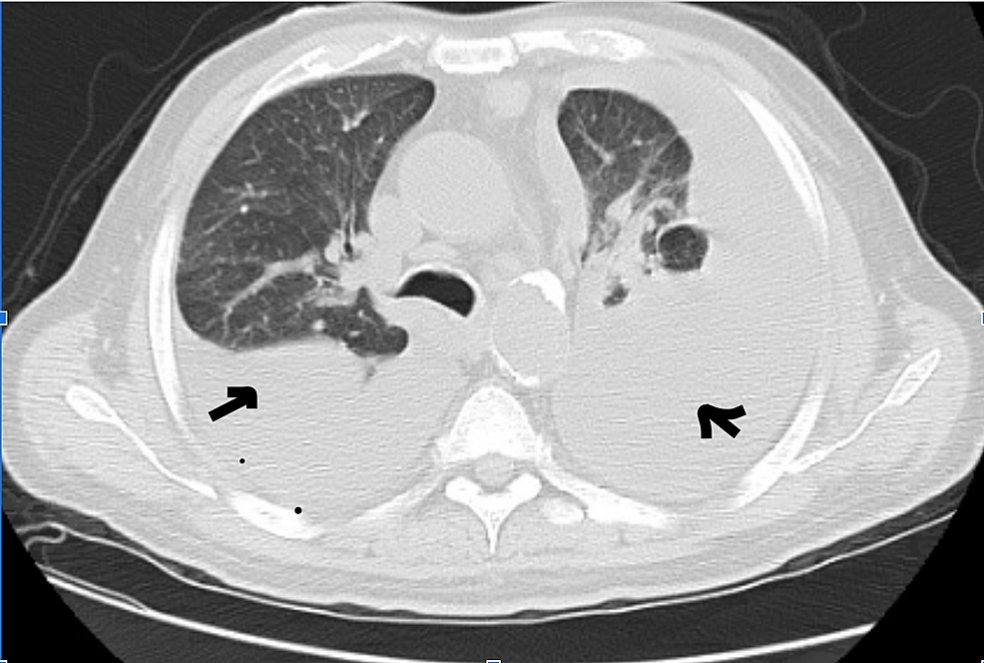
CT of the chest without contrast on admission showing bilateral pleural effusion (arrows) CT: computed tomography

A 64-year-old male with a history of hypertension, diabetes, ESRD on hemodialysis, hyperlipidemia, peripheral vascular disease, grade 2 diastolic dysfunction, and coronary artery disease with recent stent placement in the mid-left anterior descending coronary artery (two months prior to the hospital visit) initially presented to the emergency department with worsening shortness of breath for one month. Vitals in the emergency department were unremarkable except for mild tachypnea (respiratory rate of 24 breaths per minute) and desaturation to 88%, which improved to 98% with a 2-L nasal cannula. Physical examination revealed diminished breath sounds bilaterally, with bilateral one plus pitting edema of lower extremities, and jugular venous distension. EKG on admission showed normal sinus rhythm (NSR) and T wave inversion. Laboratory workup was significant for elevated troponin levels of 74.0 ng/L (normal range: 0.0-35.0 ng/l) and brain natriuretic peptide (BNP) level of 2034 pg/mL (normal range: 10-100 pg/ml). Chest X-ray showed large pleural effusions bilaterally with presumed compressive atelectasis of the lung bases (Figure 1). CT scan of the chest revealed a very large left pleural effusion with an almost complete collapse of the left lung with slight residual aeration of the left upper lobe and a moderate right pleural effusion associated with partial atelectasis of the left lower lobe with narrowing of the left upper and lower lobe bronchi, probably caused by outside compression (as shown in Figure 2).

Ultrasound-guided left-sided thoracentesis was done with the placement of a 6 French pigtail catheter, and 1000 ml of hemorrhagic pleural fluid was removed on day one. The pleural fluid analysis revealed a total cell (WBC) count of 320 per mm^3^ with 50% neutrophils. The red blood cell count was 260,000 per mm^3^. The pleural fluid protein/serum protein ratio was >0.6, serum albumin/pleural fluid albumin ratio was <1.2 g/d, confirmatory for an exudate, negative for acid-fast bacillus (AFB) smear and culture, and negative for infection or malignancy; cytology was negative (three times) and adenosine deaminase (ADA) level was 12 U/mL. Further laboratory workup showed that QuantiFERON Gold was indeterminate. Sputum for AFB stain and culture was obtained, which was negative (three times). Blood culture, vasculitis, and connective tissue workup were negative. The patient's echocardiogram did not show any pericardial fluid. He was empirically started on broad-spectrum antibiotics including vancomycin and meropenem as an underlying infectious process could not be ruled out.

**Figure 3:**
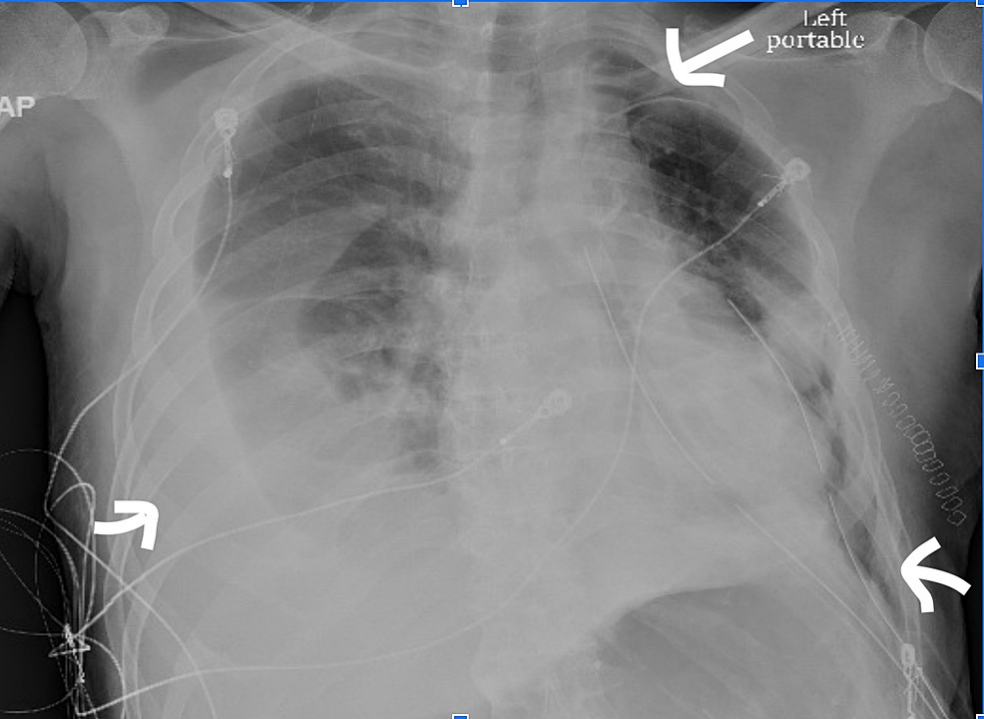
Chest X-ray after thoracotomy with two left-sided chest tubes and a small-to-moderate left pneumothorax (indicated by arrows on the right side of the X-ray) with a persistent large right pleural effusion (arrow on the left)

After reviewing the risk versus benefits of stopping dual antiplatelet therapy (DAPT) with cardiology, a decision was made to proceed with video-assisted thoracoscopic surgery (VATS) due to persistent unexplained hemorrhagic pleural effusion with a collapsed lung. DAPT was held for five days before the procedure, but notably, the red-tinged fluid (hemorrhagic pleural effusion) output from the pigtail turned into a clear fluid (non-hemorrhagic pleural effusion) output two days after stopping DAPT. Subsequent pleural fluid analysis revealed no red blood cells within the pleural fluid. However, as clear fluid drainage continued, the patient eventually underwent VATS. Subsequently, at surgery, VATS was converted to open thoracotomy, and decortication and biopsy were performed. The procedure revealed loculated pleural effusion with significant adhesions of thick fibrin peel and even possible fibrothorax. The left lower lobe was found to be completely atelectatic and unable to expand despite decortication. A post-procedure chest X-ray showed a left-sided pneumothorax with persistent large right pleural effusion (Figure 3). A total parietal pleural and visceral pleural decortication was done and sent for pathology. The biopsy report eventually confirmed fibrosis encompassing severe acute and chronic inflammation. Thereafter, a right-sided thoracentesis was performed that yielded non-hemorrhagic exudative effusion per Light’s criteria. Pleural fluid cultures and cytology were again negative. During the hospital course, the patient had a cardiac arrest, and later the family opted for comfort care.

## Discussion

A pleural effusion is defined as the collection of fluid in the pleural space. Normally, the equilibrium of pleural fluid due to production and absorption is maintained by the visceral and parietal pleura. Any disruption in the homeostasis results in the development of pleural effusion. It has been shown that the mean rate of both the production and the absorption of pleural fluid is about 0.2 mL/kg/hr, which means that the entire volume of the pleural fluid normally turns over in one hour [3]. Low oncotic pressure, elevated pulmonary capillary pressure, increased permeability, lymphatic obstruction, and diminished negative intrapleural pressure are some of the mechanisms that contribute to pleural effusions [3]. A pleural effusion is further categorized into either transudative or exudative according to the Light's criteria [4]. The most common causes of bilateral pleural effusions are thought to be congestive heart failure (CHF) and renal or liver failure. ESRD patients on dialysis should be assessed for the quality and efficiency of the dialysis. Under-dialyzed patients are overhydrated and can have pleural effusion. In the setting of bilateral effusions, however, exudative effusions are more common than transudative effusions, and malignancy is the most common cause of an exudative unilateral effusion [5].

A hemorrhagic effusion is a type of exudative pleural effusion that is defined as an erythrocyte count of greater than 100,000 cells/µl. Some of the causes of hemorrhagic pleural effusions are outlined in Table 1. The difference between a traumatic tap and a hemorrhagic pleural effusion is that in a traumatic tap, there is clearing up on the subsequent sampling of pleural taps. On the other hand, hemothorax occurs when the pleural fluid hematocrit is >50% of the serum hematocrit [5]. In our case, spontaneous bilateral hemothoraces were observed in a dialysis patient who was recently started on aspirin and Plavix due to cardiac stent placement. After the discontinuation of DAPT, the hemothoraces resolved spontaneously, which was confirmed by repeat thoracentesis from the right-sided pleural effusion.

**Table 1:** Causes of hemorrhagic pleural effusion SLE: systemic lupus erythematosus

Causes	Examples
Infection	Bacterial pneumonia, Mycobacterium tuberculosis
Malignancy	Pleuropulmonary malignancy, bronchogenic carcinoma, leukemia (acute and chronic), pleural tumor lymphoma, e.g., Hodgkin's lymphoma, non-Hodgkin's lymphoma. Tumors of the ribs, e.g., osteosarcoma Metastasis to pleura and mediastinal nodes, e.g., breast carcinoma, choriocarcinoma, malignant melanoma, hypernephroma, retroperitoneal chondrosarcoma. Bony tumor, e.g., Ewing sarcoma
Connective tissue disease	SLE
Asbestos exposure	Benign as well as malignant mesothelioma
Abdominal diseases	Pancreatitis ovarian tumors - benign (Meigs syndrome as well as malignant tumors, uremic pleuritis, diaphragmatic hernia)
Cardiovascular	Aneurysm rupture, pulmonary infarction, pulmonary thromboembolism post coronary artery bypass grafting
Bleeding disorder	Overdose of anticoagulant, thrombotic microangiopathies, Thalassemia, liver cirrhosis
Miscellaneous causes	Superior vena cava syndrome, Kawasaki disease, chronic renal failure, and intralobar sequestration

The occurrence of a hemothorax can be either acute, likely due to sharp or blunt chest trauma leading to vascular injury, or spontaneous. Spontaneous hemothorax can occur due to anticoagulation therapy, vascular malformations, inherited coagulopathies, bleeding from systemic vessels (aortic dissection, ruptured patent ductus arteriosus, leaking internal mammary artery aneurysm), bleeding from pulmonary vessels (arteriovenous malformations), active tuberculosis, and sub-diaphragmatic causes (endometriosis, splenic artery aneurysm) [6]. Therapeutic anticoagulation is one of the most common causes of a hemothorax. Spontaneous hemothorax in patients on anticoagulation therapy has also been reported in 14 cases [7]. The management of hemothorax is mainly focused on the etiology (acute vs. spontaneous). The management of hemothorax in most cases is close observation and may sometimes require the placement of a chest tube for drainage. However, if a hemothorax is retained, it increases the likelihood of complications such as chronic empyema and fibrothorax. Similarly, in our case, the patient developed hemothorax after starting DAPT, which was complicated by the development of a fibrothorax that eventually required emergent decortication.

Bleeding is not an uncommon risk associated with long-term anticoagulation and antiplatelet therapy. DAPT, consisting of aspirin with clopidogrel, remains the cornerstone of treatment in patients with major ischemic events, despite the bleeding risk. Based on the current literature, DAPT shall be employed for 12 months after percutaneous coronary intervention (PCI) vs. six months in patients who are at increased risk of bleeding. The use of DAPT in patients with renal failure who already have platelet dysfunction increases the risk of bleeding [8-12]. As we all know, owing to platelet dysfunction and changes in the interaction between platelets and walls of the vessel, the risk of bleeding is usually high in patients on hemodialysis [13]. Other minor factors leading to a bleeding tendency are the presence of anemia, the accumulation of medications, and anticoagulation use during dialysis. Dialysis helps to mitigate the risk of bleeding in ESRD patients by removing the uremic toxins but does not eliminate them [14]. Many cohort studies have shown that the risk of bleeding in hemodialysis patients on an antiplatelet agent is not increased while many other studies have shown that the bleeding risk does increase. One extensive meta-analysis showed that the risk of bleeding is increased in hemodialysis patients taking dual antiplatelet agents while there was no increase in bleeding risk in hemodialysis patients taking a single antiplatelet agent [15-17]. In our case, the patient did not have any trauma, infection, or malignancy; however, he had undergone a recent cardiac catheterization due to non-ST-elevation myocardial infarction (NSTEMI), after which DAPT was started. Two months post-cardiac catheterization, the patient presented with spontaneous symptomatic bilateral pleural effusions with thoracentesis suggestive of hemorrhagic pleural effusions, which was likely secondary to the use of DAPT in our patient with a history of chronic hemodialysis with platelet dysfunction and changes in the interaction between platelets and walls of the vessel. This association is quite rare and, to our knowledge per our literature review, only a single case has been reported with similar findings [18].

## Conclusions

It is imperative to weigh the risks and benefits of DAPT in all individuals, especially in high-risk individuals as in our case: an elderly male patient with ESRD. DAPT is associated with potential risks of bleeding; however, our case reveals that it can also result in unusual and rare conditions such as bilateral hemorrhagic pleural effusions. We suggest that clinicians should be cognizant of the possibility of these agents causing bilateral pleural effusions, especially when such patients are on dialysis, and offending agents should be promptly discontinued once identified. This in turn will help prevent further complications as found in our patient. Further studies are needed to verify whether a statistically significant association exists between DAPT use and the development of bilateral pleural effusions. This will enable us to formulate guidelines to diagnose and treat such patients in the future.
